# A Retrospective Cohort and Systematic Review of Non-Operative Management of Exposed Calvaria Post-Radiotherapy

**DOI:** 10.7759/cureus.8751

**Published:** 2020-06-21

**Authors:** Pinkal Patel, James Edward Massey Young, Mark McRae, Jenny Santos, Carolyn Levis, Michael K Gupta, Sophocles Voineskos, Lucas Gallo, Emily Dunn, Matthew C McRae

**Affiliations:** 1 Pediatrics, McMaster University, Hamilton, CAN; 2 Otolaryngology—Head and Neck Surgery, McMaster University, Hamilton, CAN; 3 Plastic Surgery, McMaster University, Hamilton, CAN; 4 Surgery, Division of Plastic Surgery, McMaster University, Hamilton, CAN; 5 Plastic Surgery, St. Joseph's Hospital, McMaster University, Hamilton, CAN

**Keywords:** radiation, exposed calvaria, radiotherapy, non-operative management, osteoradionecrosis

## Abstract

Scalp defects with exposed calvaria that have previously been irradiated present a unique reconstructive challenge. Patients with previously radiated scalp defects often have few reconstructive options due to poor health or personal choice. The aim of this study was to evaluate the results of non-operative management for patients with prior radiotherapy to the scalp who developed exposed calvaria. The outcomes of interest were major and minor complications related to exposed calvaria with a time frame of follow-up of greater than one year or death from any cause.

A retrospective chart review was performed to identify patients with prior radiotherapy and surgery for skin cancer to the scalp who subsequently developed exposed calvaria. Data from four surgeons from 2008 to 2019 was collected. Next, a systematic review of PubMed, EMBASE, Cochrane Library, and CINAHL was conducted to identify articles in which non-operative management was utilized for exposed calvaria post-radiotherapy.

Nineteen patients were identified who received radiotherapy either before developing recurrent malignancy requiring operation or requiring radiation postoperatively because of close or involved margins and who subsequently developed exposed calvaria. Six of these patients had an additional attempt at local flap or skin grafting that failed. All patients had an American Society of Anesthesiologists score of three or four. All were managed with local wound care. Ten patients had near-complete healing with wound care alone. Eight patients are still alive from one to six years after the presentation. One patient, who remains alive, developed an intracranial abscess requiring long-term antibiotics but was medically compromised by concomitant myelodysplastic syndrome, mantle cell lymphoma on chemotherapy, atrial fibrillation on anticoagulation, and heart failure. Three patients developed new malignancies requiring re-operation with watchful waiting. Two of the three cases resulted in failure to control disease, but control of malignancy occurred in one case with resection of recurrent cancer and exposed bone.

The systematic review of the literature yielded three studies that met the inclusion criteria. None of the studies encountered cases of meningitis, encephalitis, or death due to the non-operative treatment of exposed calvaria post radiation.

Coverage of the calvaria with well-vascularized tissue is the reconstructive goal in the majority of circumstances. This case series and systematic review found that non-operative management of exposed calvaria post-radiotherapy can be an option for patients who are either not candidates for aggressive surgical treatment or who refuse surgery.

## Introduction

Options for scalp reconstruction after skin cancer excision include healing by secondary intention, primary closure, tissue expansion, skin grafting, local flaps, and free tissue transfer [1-2]. Prior radiotherapy to the scalp limits viable reconstructive options. While the pericranium of non-radiated scalps can be skin grafted, the pericranium if irradiated is no longer well vascularized and often fails to provide adequate blood supply for healing [3]. Scalp tissue inherently has limited elasticity and after radiotherapy, local flaps become even more difficult, and sometimes impossible, due to microvascular occlusion and fibrotic changes. Radiated scalp tissue is not easily moved and has decreased wound healing potential [4]. Free tissue transfer is possible when all local and regional options are unsuitable; however, these surgeries require a general anesthetic and can take many hours to perform. Central lesions over the sagittal sinus carry the risk of major blood loss as the irradiated bone and fibrosis underlying dura make resection of the bone extremely treacherous. In some patients with multiple medical comorbidities, any prolonged operation may not be a viable option.

Secondary intention healing is the least invasive wound management technique for small scalp wounds and may be appropriate for patients who are poor surgical candidates [2]. However, there are concerns related to the longer duration of healing and exposure of the scalp calvaria, such as osteomyelitis and infection. Secondary intention healing of the scalp may not occur in the setting of prior radiation therapy due to osteoradionecrosis of the bone. Poor blood supply to the area of exposed bone can potentially lead to serious complications progressing from osteomyelitis to meningitis and brain abscess.

The purpose of this retrospective study and systematic review is to evaluate the results of non-operative management of exposed calvaria in patients with prior radiation therapy. This is meant to address a clear gap in the scientific literature. These patients, in a process of shared decision making with their surgeon, decided against complex reconstruction or were deemed too high operative risk for the procedure. The outcomes of interest were major and minor complications related to exposed calvaria with a length of follow-up of greater than one year or until death from any cause.

Preliminary results of this study were presented as a podium presentation at a conference, the abstract of which was later published in the journal "Plastic and Reconstructive Surgery-Global Open" [5]. 

## Materials and methods

Retrospective chart review

A retrospective chart review was performed on a cohort of patients who developed exposed calvaria with a history of radiation treatment to the area. Data from patient records from four surgeons at McMaster University from 2008 to 2019 was collected with all patients fitting criteria included. Patient data were extracted from electronic medical records. The inclusion criteria consisted of male or female patients of any age who developed exposed calvaria due to treatment of skin malignancy who had radiation therapy to the scalp either remotely or for primary skin cancer treatment. None of the patients had known metastasis at the time they were seen by the surgeon for exposed calvaria. The exclusion criteria consisted of patients who had incomplete records, preventing knowledge of the type of treatment they received or complications experienced, no exposed calvarial bone, and no prior radiotherapy to the scalp. The study was approved by the Hamilton Integrated Research Ethics Board (HiREB #3077C).

Data extraction and analysis

Extracted data included patient demographics, number and type of co-morbidities, the American Society of Anesthesiologists (ASA) class, type of skin cancer, radiation history, wound characteristics, type of management, and wound complications. Minor wound complications were soft tissue infection and osteomyelitis treated as an outpatient. Meningitis, dural abscess, and encephalitis were considered major wound complications. The ASA class was determined by the anesthesiologist to assess the patient’s preoperative physical status. The defect size was calculated by the diameter of the exposed bone.

Systematic review

A systematic review was performed in accordance with the guidelines proposed by the Preferred Reporting Items for Systematic Reviews and Meta-analyses (PRISMA) statement [6]. An electronic search of bibliographic databases (PubMed, EMBASE, Cochrane Library, Cumulative Index to Nursing and Allied Health Literature) using keywords related to skull, scalp, wound, non-operative, skin cancer and radiotherapy. Medical subject headings, including wounds and injuries/therapy, osteoradionecrosis/therapy, osteoradionecrosis/complications, head and neck neoplasms/therapy, were employed when possible (Appendix Table 1). A search of unpublished, ongoing trials (World Health Organization International Trials Registry Platform, Web of Knowledge Conference Proceedings, and ClinicalTrials.gov) was conducted using the following keywords; “exposed skull,” “skin neoplasm,” “skull,” “scalp,” or “calvaria/um”. The reference lists of the selected articles were assessed for additional studies of relevance. This systematic review was registered at PROSPERO (registration number CRD42017084753; link http://www.crd.york.ac.uk/PROSPERO/display_record.php?ID=CRD42017084753).

Study selection

Two reviewers (P.S.P. and J.S.) independently screened and identified relevant articles to include in the full-text screening. Consensus on article inclusion was verified with a third reviewer (M.C.M.) The inclusion criteria included articles that 1) considered non-operative management for exposed calvaria in patients’ post-radiotherapy for skin malignancy; 2) included patients of any age; 3) were published between Dec 31, 1985 and Dec 31, 2017; 4) were of any study design, including case reports; and 5) were human subjects. The exclusion criteria consisted of 1) surgical management of exposed calvaria; 2) lack of radiotherapy to the scalp; 3) malignancy other than squamous cell carcinoma, basal cell carcinoma, or melanoma; and 4) non-English language articles.

Data extraction

Data extraction was performed independently by one reviewer (P.S.P.) and verified by a second reviewer (J.S.). The following data were extracted from relevant articles: sample size, sex, age, type of skin cancer, history of radiotherapy, type of management, follow-up period, and complications (including soft tissue infection, osteomyelitis, meningitis, and encephalitis). Disagreements were resolved through discussion until consensus was achieved, and consultation with an arbitrator (M.C.M.), if needed.

Risk of bias assessment

The risk of bias was assessed independently and in duplicate by two reviewers (P.S.P and L.G.). Disagreements were resolved through discussion until consensus was achieved, and consultation with an arbitrator (M.C.M.), if needed. As all included studies were non-randomized, the Newcastle-Ottawa scale was selected for methodological assessment. This instrument evaluates studies by allotting points in terms of selection, comparability, and outcomes. To fulfill the requirement of adequate follow-up of cohorts in the outcome section, 80 percent was chosen as a minimum adequate follow-up.

## Results

Retrospective chart review

From the practices of four surgeons, 19 patients were identified who had a prior history of radiation therapy to the scalp and developed exposed calvaria (Table 1).

**Table 1:** Results of non-operative treatment of previously radiated exposed calvaria M: male, F: female, SCC: squamous cell cancer, BCC: basal cell cancer, MM: malignant melanoma, MFH: malignant fibrous histiocytoma

	Sex	ASA class	Pathology	Scalp defect size in cm diameter	Defect final follow-up	Age at diagnosis	Age at death or last follow-up	Final follow-up
1	M	4	SCC	25	Larger due to 2 surgical attempts at closure for recurrence	91	94	Deceased: subdural hemorrhage after a fall
2	M	4	BCC	16.5	Clean, smaller, asymptomatic	85	88	Deceased: abdominal crisis
3	F	4	SCC	18	Multiple operations and craniectomy for recurrences	85	88	Alive with local and brain recurrence
4	M	3	SCC	9.6	Unchanged but developed Marjolin SCC of the scalp at 79	73	81	Deceased: recurrent scalp cancer to brain
5	M	3	MM	5	Asymptomatic & smaller	93	95	Deceased: lip cancer
6	M	4	SCC	20	Smaller with progressive healing	81	86	Deceased: aspiration pneumonia
7	M	4	SCC	6	Stable, unchanged	84	87	Deceased: cardiac arrest
8	M	3	SCC	7	Progressive healing with small residual defect	88	94	Alive and well
9	M	4	SCC	Not recorded	Bone debrided at 88, progressively smaller	83	90	Deceased: renal failure
10	M	4	MM	2	Healed	78	80	Alive and well with scalp healed
11	M	4	SCC	4	Smaller in size	81	82	Deceased: eyelid cancer
12	M	3	SCC	9	Healed then developed new cancers on scalp requiring resection	77	79	Alive and well with scalp healed
13	M	4	SCC	8	Developed extradural abscess with slow healing	84	86	Alive but with ongoing infections and new cancer of the scalp
14	M	4	SCC	15	<1 cm, almost fully healed	76	78	Alive and well
15	M	3	SCC	1	Slow healing, half of original size	75	76	Alive and well
16	F	4	MM	12	Unchanged but recurrent adjacent tumor resected	73	76	Alive, on chemotherapy for local and systemic melanoma
17	F	4	SCC	16	Recurrent tumor in exposed bone	84	86	Deceased: inoperable tumor into the sagittal sinus
18	M	4	MFH	4.5	Stable, unchanged	83	85	Deceased: cardiac disease
19	M	3	MM	4 in multiple sites	Smaller in size	74	82	Deceased: abdominal sepsis

There were no formalized criteria based on comorbidities for non-operative treatment used by the surgeons. Sixteen were male and three were female with a mean age of 81.5 years and standard deviation (SD) of 5.7. All patients received the previous radiotherapy for biopsy-confirmed skin cancers of the scalp or post-operative radiotherapy for positive or close margins after primary surgery. Most patients had a high number of comorbidities declared in the initial consultation (mean = 7.7, SD = 3.8). All patients with exposed calvaria post-radiation therapy by definition had a degree of osteomyelitis/osteoradionecrosis as they had previously irradiated bone that failed to heal over a period of greater than three months [7]. Approximately 32% of patients had diabetes, 26% with coronary artery disease, 26% with pulmonary disease, 84% had dyslipidemia, 100% were hypertensive, and 63% were former smokers. There were no solid organ transplant patients. Only one of the 19 patients had a previous free tissue transfer attempted for calvaria coverage. Many patients were on anticoagulation for atrial fibrillation and other reasons. All patients were followed on by their surgeon with debridement with forceps and a rongeur and local or systemic antibiotics as needed. All had daily or biweekly dressing changes by home care nursing staff or family members. Local complications were very rare. One patient (patient 4) developed what we presume was a new Marjolin ulcer six years after presenting with exposed bone. The patient developed further recurrence intracranially despite re-operation and additional radiation therapy. Only one patient developed an intracranial infection (pt 13) that stabilized with long-term antibiotics. The patient remains medically stable despite chemotherapy for mantle cell lymphoma and myelodysplastic syndrome. Causes of death in this group of older, medically compromised patients included gastric volvulus, aspiration pneumonia and bacteremia, bowel obstruction, recurrent lip SCC, and cardiac disease.

Case examples

**Figure 1:**
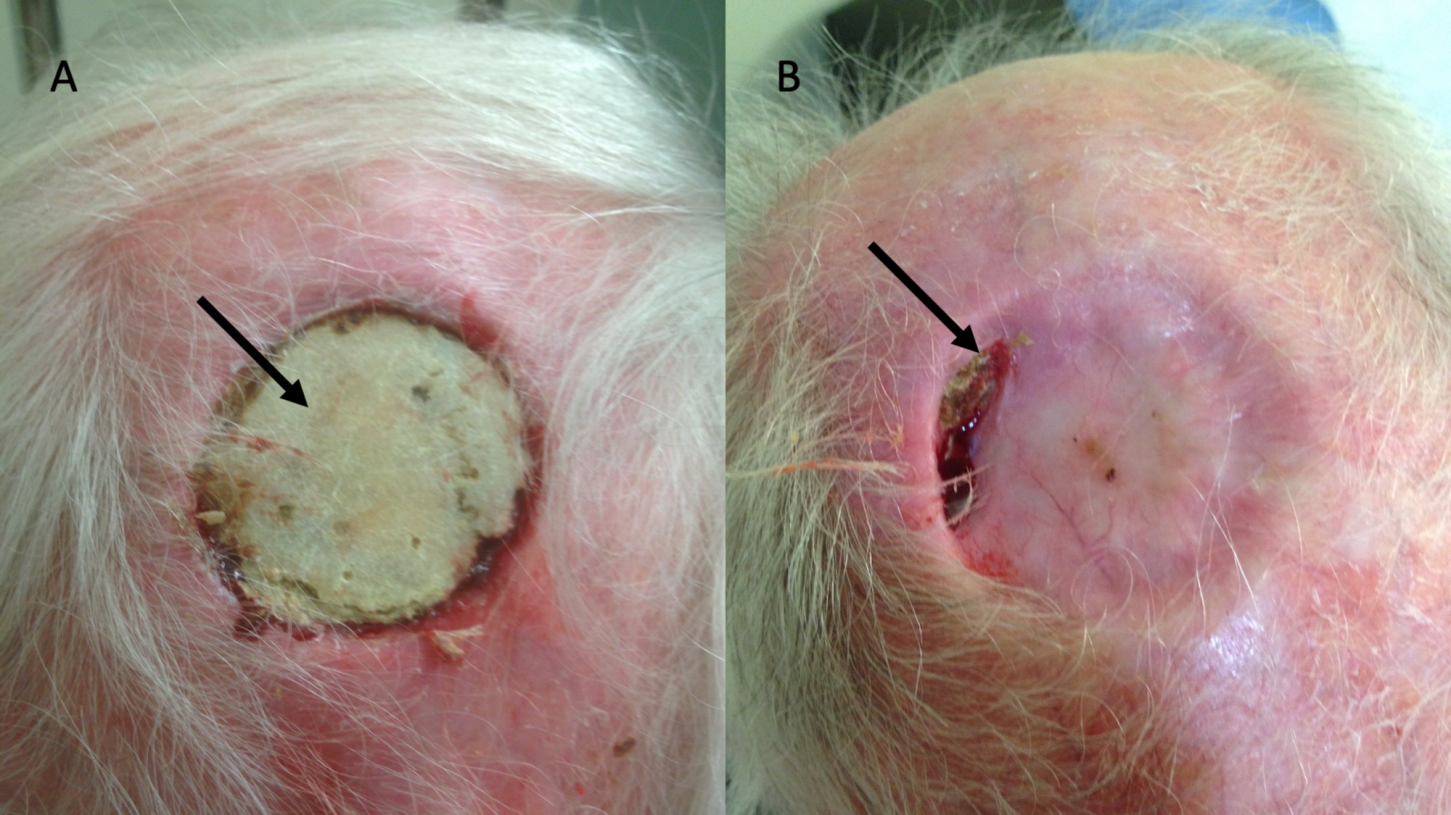
Patient 8 A) on presentation in 2013 post radiation and surgery with the arrow pointing to exposed radiated calvaria; B) demonstrating healing of previously radiated exposed calvaria over a time course of 7 years Arrow points to a small asymptomatic area of exposed bone that remains.

Patient 8 presented in 2013 with exposed calvaria following the operative excision of skin cancer and radiation therapy (Figure 1A). A photo taken seven years later in 2020 shows a small residual area with an asymptomatic area of exposed bone. There are new radiation changes due to other skin cancers of the scalp treated in the interim (Figure 1B).

**Figure 2:**
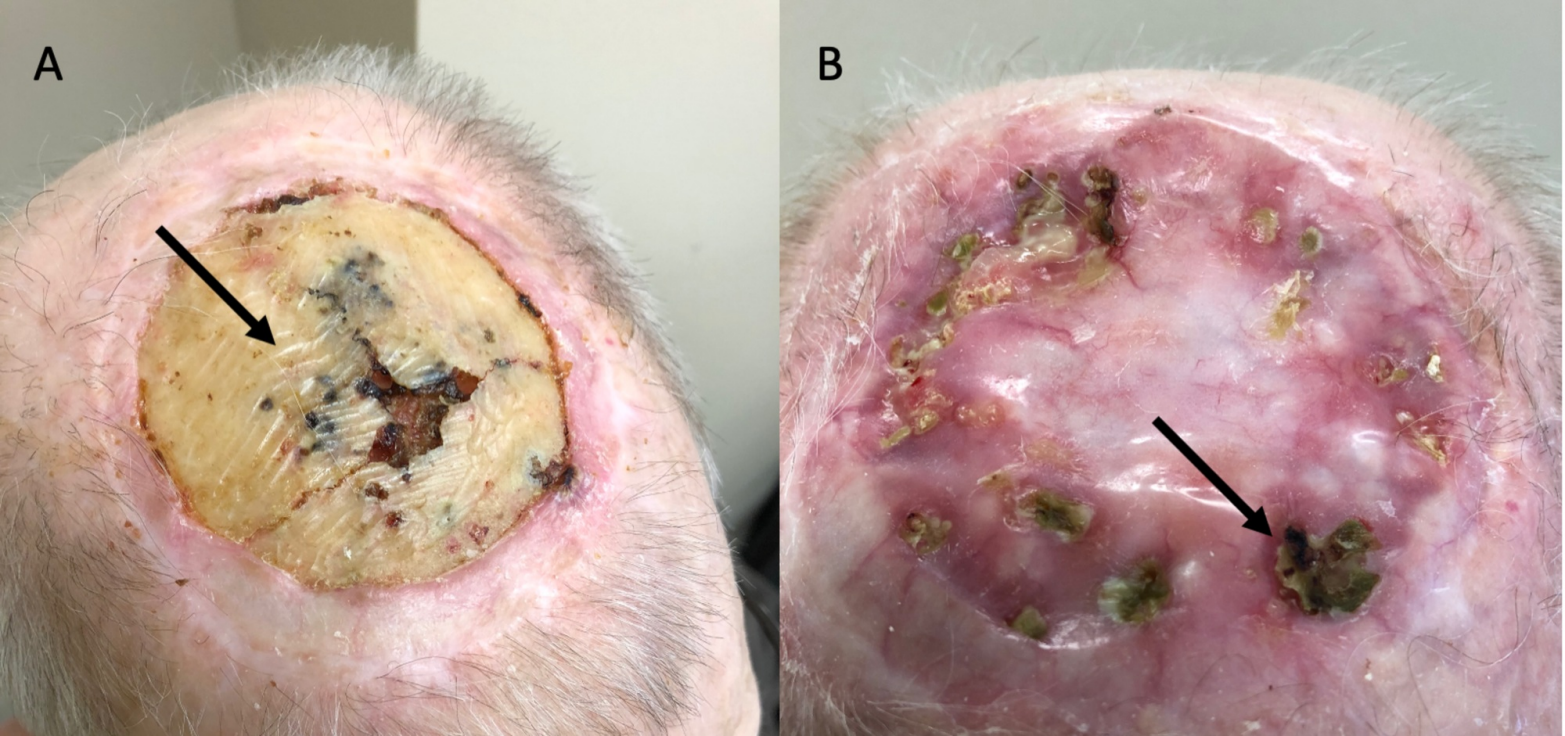
Patient 14 A) on presentation in 2017 after failed skin graft, arrow pointing to burred outer cortex of radiated calvaria; B) 3 years later with progressive epithelialization with arrow pointing to one of the multiple small areas with remaining exposed bone

Patient 14 presented in 2017 with recurrent squamous cell cancer to the scalp post radiation therapy. The tumor was resected including periosteum. The outer cortex of the skull was burred to improve the chance of skin graft healing. Post-operative infections resulted in total skin graft loss, which resulted in a large area of exposed bone (Figure 2A). A photo taken from March 2020 shows near-complete healing with small areas at the edge that have yet to epithelialize (Figure 2B). 

Systematic review

**Figure 3:**
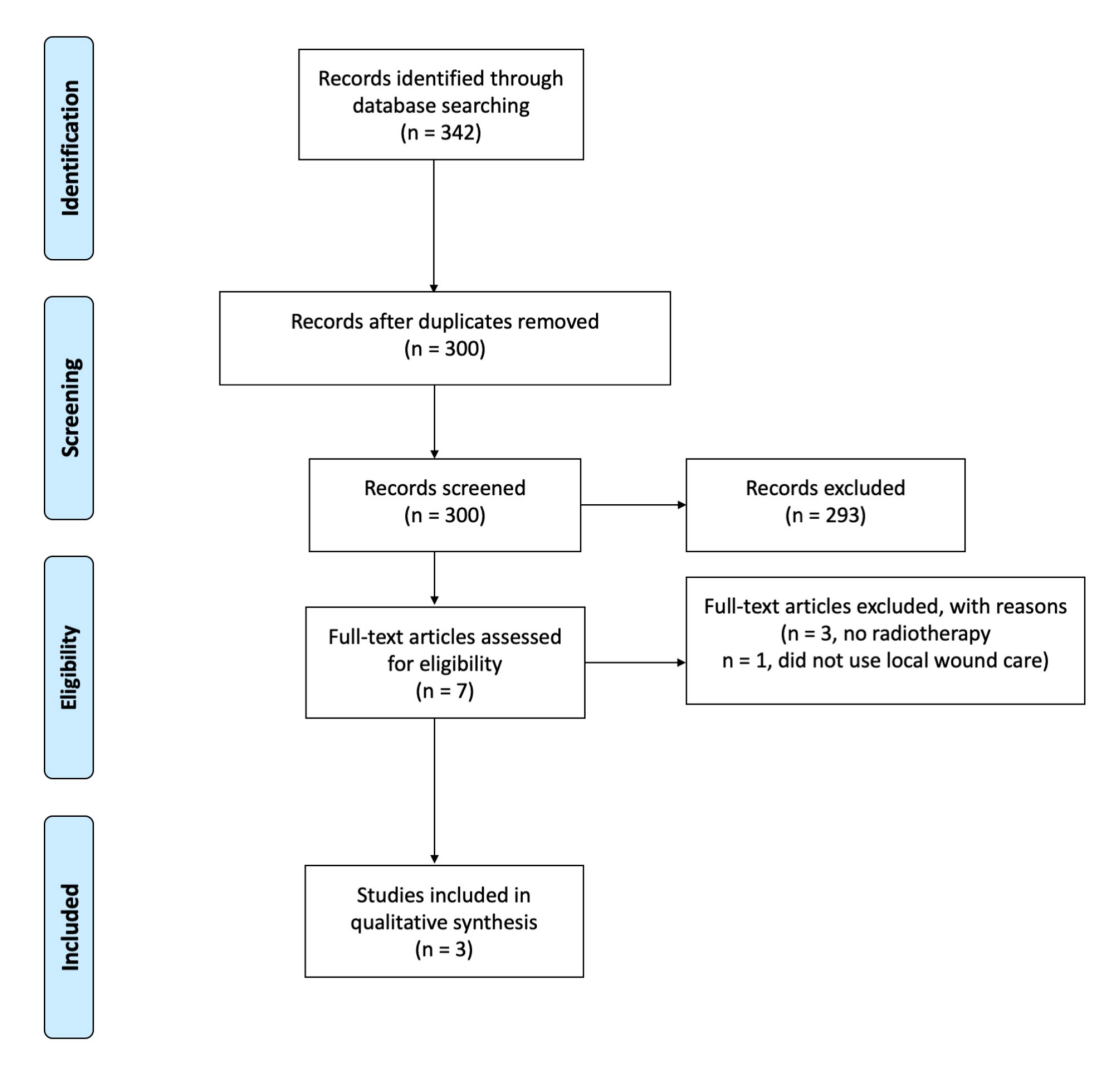
Systematic review PRISMA flowchart

The electronic search of databases yielded 342 articles. After reviewing titles and abstracts, seven articles were considered for full-text review, of which three met the inclusion criteria (Figure 3) [8-10]. 

All studies were retrospective in nature, including one cohort study, one case series, and one case report. Table 2 summarizes the characteristics of the included studies. Data from a total of 24 patients across the three studies were reviewed, with follow-up ranging from 7 months to 6 years.

**Table 2:** Characteristics of studies included in the systematic review M: male, F: female, SCC: squamous cell cancer, XRT: radiation therapy [8-10]

Reference	Study design	Sample size	Age/Sex	Type of skin cancer	XRT to scalp?	Management	Complications	Follow-up
Beroukhim et al. (2014)	Case report	1 patient	76 / M	SCC	Yes	Non-operative debridement, soap, and water daily, cover wound with petroleum jelly & non-adherent dressing x 3 years, subsequent use of 0.5% topical timolol BID to wound x 4 months.	None noted with regards to exposed calvarium, contact dermatitis in response to 0.5% timolol application	3 years and 4 months
Snow et al. (1994)	Retrospec-tive cohort review	91 patients in total but only 21 that meet inclusion criteria	Mean age 72 / 57 M and 34 F	Not specified	24 of 91 patients or 26%	Bedside bone debridement every 3 weeks, daily soap and water followed by antibiotic ointment, and nonadherent dressing	Soft-tissue infections (3/112 or 2.7%); osteomyelitis (0); meningitis (0)	7 months
Lloyd et al. (2016)	Case series	3 patients total, but only 2 that meet inclusion criteria	Not specified	SCC	Yes	Wash with soap and water, apply petrolatum ointment daily, debridement of loose necrotic bone prn x 6 years post-radiation	Wound infections (3/3); osteomyelitis (0); cranial abscess (0); meningitis (0)	6 years

Included studies

Beroukhim and Rotunda describe a 76-year-old male patient who had Mohs surgical excision without reconstruction for recurrent squamous cell carcinoma (SCC) of the scalp, previously treated with excision and radiation therapy. After three years of wound care, topical 0.5% timolol maleate was used for hypothesized efficacy at inducing epithelialization in a refractory surgical wound. The entire wound completely re-epithelialized within four months. The authors did not report complications such as local soft tissue infections, osteomyelitis, encephalitis, or meningitis occurring [8].

Snow et al. conducted a retrospective chart review of 91 patients (112 wounds) who underwent non-operative management of exposed scalp or facial bone after Mohs surgery for skin cancer. Of the 91 patients, only 24 received prior radiotherapy for skin cancer treatment. The authors report soft tissue infections in 2.7% or three of the 112 wounds and zero cases of osteomyelitis or meningitis. Data for the patients who received prior radiotherapy could not be separated from the patients without radiation therapy exposure [9].

Lloyd et al. reported on three patients who developed osteonecrosis of the cranium after treatment of SCC with Mohs surgery and radiation therapy. All three patients elected to undergo non-operative management. After six years, one patient underwent definitive microsurgical reconstruction. The authors note that soft tissue infections occurred in all three patients despite wound care, and these were managed with antibiotics [10].

Risk of bias assessment

The overall quality of methodology in the non-randomized studies was moderate, with scores ranging from five to six stars (Figure 2). Generally, the studies did well in terms of outcome domains but lost points in patient selection and comparability domains. 

**Figure 4:**
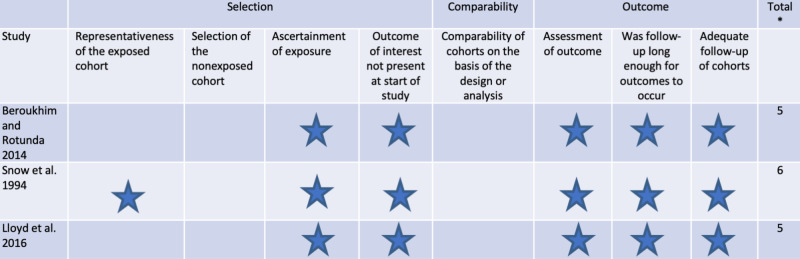
Risk of bias of the included studies using Newcastle-Ottawa tool for assessing the risk of bias The highest score a study can achieve is 9 if there was a comparison group and 6 if there was no comparison group. [8-10]

## Discussion

In our progressively aging population, there is an increasing number of patients with skin cancers of the scalp requiring radiation therapy. Recurrence after radiation involves the assessment of the patients' overall medical condition, which may inform options for reconstruction. Retrospective evaluation of our patients suggests that in reconstructing a new skin cancer defect in a previously radiated scalp, particularly when the depth of margin involves periosteum, vascularized rotation flap is the first-line treatment and can avoid subsequently exposed bone. A rotation flap may require revision of the standing cone deformity in a subsequent stage but adds minimally to the procedure length and would prevent the need for long-term wound care.

The decision to undergo free tissue transfer to restore soft tissue coverage overexposed and previously radiated calvarium is straightforward in a patient who is physically fit enough to undergo the surgery without the significant threat of perioperative or intraoperative morbidity. However, there is a hesitancy in performing complex reconstruction such as free tissue transfer surgery in some patients, and indeed, not all patients or their caregivers consent to the procedure.

Our study population of 19 patients could be defined as “elderly” with a mean age of 81.5 and ASA class of either III or IV. Age should not be used as a deciding factor to determine which patients are suitable candidates for major surgery. Rather, it is more likely that with increasing age, patients acquire a greater number of co-morbid conditions including hypertension, coronary artery disease, pulmonary disease, and diabetes [11-13]. For this patient population, reconstructive surgeons should employ shared decision making with the medical care team of other physicians and with the patient in order to decide on an optimal treatment strategy.

While surgical management can pose significant risks, non-operative management is also burdensome. A dressing regimen that keeps the calvarium and surrounding soft tissue clean, moist, and protected can be quite onerous on the patient as well as the health care system. This is combined with evaluation by a physician at regular intervals to ensure that soft tissue infections are properly addressed with local or systemic antibiotics. This study found that secondary intention healing can occur even in the setting of radiation [9]. Of interest, 10 of the 19 patients, some with large areas of the exposed calvaria, went on to near-complete healing of their scalp wounds over the prolonged time period of follow-up. These patients need regular evaluation to rule out new or recurrent disease. There should be a low threshold for skin biopsy and bedside removal of outer table necrotic bone fragments that can be sent for pathologic examination. 

The aim of this study was to determine the results of non-operative management for radiated exposed calvaria. The ideal patient groups to compare would be those who undergo reconstruction and those who undergo non-operative management in a randomized trial. This study design is not ethically tenable as immediate closure of a skin defect over the bone is performed whenever possible. There were three patients that required re-operation for malignancy. One failed on re-treatment (patient four). Another required re-excision and four operations to achieve coverage (patient 12). A third patient had two additional new or recurrent cancers of the scalp that were resected with attempts at closure that failed leading to a larger area of the exposed calvaria. 

The main finding of this study is that most patients did remarkably well with non-operative management. Although there was a high infection rate, these were adequately managed with close follow-up and treatment with antibiotics. Eleven patients have died in the follow-up period as would be expected of the age demographics. The cause of death was not directly attributable to the exposed calvaria although one patient had an SCC that most likely represented a Marjolin tumor and failed re-treatment. 

The systematic review was conducted in hopes of determining whether complications have been reported in the literature. There were no cases of osteomyelitis or any major complications such as meningitis and encephalitis recorded. The findings of the systematic review support our clinical findings that major complications with non-operative treatment are indeed quite rare. 

A limitation of the present study is the selection bias inherent in its retrospective design. We are also limited by sample size, which may not capture instances of rare complications.

Based on this retrospective cohort study and systematic review, we have generated the following treatment algorithm. Patients who have had radiotherapy to the scalp and subsequently develop exposed calvaria may be considered for non-operative management if the following three criteria are met:

1. A minor procedure such as a local flap or skin grafting is not possible or not successful

2. The patient would have difficulty undergoing a prolonged general anesthetic

3. The patient or their guardian do not wish to undergo free tissue transfer

An important point to underscore is that any chronic wound, particularly one that has been previously treated for skin cancer with radiation or surgery, could represent local recurrence. This occurred in four patients in our study (patients 3,4,16,17). The excision of recurrence in the skin can often be treated with excision under local anesthetic with minimal morbidity, while bone involvement portends a worse prognosis. Worsening wounds should undergo biopsy.

The morbidity of surgery and post-operative recovery must be balanced with the complications of non-operative management. For the patients included in this cohort, the decision was made to not perform complex reconstruction. Based on this limited evidence, we suggest that local wound care may be a reasonably safe treatment option when reconstruction is not possible.

## Conclusions

Exposed calvaria that has previously been radiated is a reconstructive challenge. For some patients with exposed calvaria that can only be covered with complex reconstruction such as free tissue transfer, a lengthy operation may not be safe. This retrospective study and systematic review found that non-operative management of exposed calvaria in those with prior radiotherapy can be a safe option for patients who are either not candidates for surgical treatment or who refuse surgery. This is a viable option in the reconstructive algorithm of the radiated scalp as there are cases where a non-operative treatment strategy is safe and necessary. 

## Appendices

**Table 3:** Systematic review search strategy

Databases	Search Strategy
PubMed, EMBASE, Cochrane, CINAHL, Web of Knowledge Conference Proceedings, ClinicalTrials.gov	1- skull
	2- calvarium
	3- 1 or 2
	4- scalp wound
	5- scalp defect
	6- 4 or 5
	7- non-operative treatment
	8- non-operative management
	9- healing by secondary intention
	10- 7 or 8
	11- 3 or 6 and 10
	12- skin cancer
	13- skin malignancy
	14- skin neoplasm
	15- 12 or 13 or 14
	16- 3 or 6 and 15
	17- Radiotherapy
	18- 3 and 9 and 15
	19- 3 and 15
	20- Dressings
	21- 15 and 20
	22- osteoradionecrosis
	23- osteoradionecrosis/therapy [MeSH]
	24- osteoradionecrosis/prevention and control [MeSH]
	25- osteoradionecosis/complications [MeSH]
	26- wounds and injuries/therapy [MeSH]
	27- head and neck neoplasms/therapy [MeSH]
